# From career exploration to experiencing well-being: the psychological mechanisms of university students’ career development

**DOI:** 10.3389/fpsyg.2026.1807206

**Published:** 2026-05-20

**Authors:** Yue Dong, Sheng Lin, Xiaoxia Huang

**Affiliations:** School of Internal Programs, Guangdong University of Finance, Guangzhou, Guangdong, China

**Keywords:** career adaptability, career exploration behavior, perceived control, subjective well-being, university students

## Abstract

**Background and Objective:**

Guided by career construction theory, this study investigated the association between career exploration and subjective well-being among university students, with a focus on the roles of career adaptability and perceived control.

**Methods:**

A convenience sample of 817 students participated in an online survey, and all constructs were measured using validated instruments, including the Career Exploration Behavior Scale, Career Adapt-Abilities Scale, Happiness Index Scale, and Sense of Control Scale.

**Results:**

Results from structural equation modeling indicated that career exploration was positively associated with subjective well-being, and that career adaptability partially mediated this relationship. In addition, perceived control moderated the association between career exploration and career adaptability, with a stronger link observed among students reporting higher levels of perceived control.

**Conclusion:**

These findings underscore the relevance of promoting career exploration, enhancing career adaptability, and supporting perceived control to foster students’ subjective well-being, offering both theoretical reinforcement for career construction theory and practical insights for career development initiatives in higher education.

## 1 Introduction

University students are transitioning from campus life to professional development and encounter various challenges, including workplace competitiveness and future career planning. During this period, subjective well-being (SWB) is particularly important, as it is directly related to the pursuit of meaningful career goals (Ruan et al., 2025; Zhang et al., 2022). In today’s rapidly changing social and occupational environment, students often experience uncertainty and pressure when making career-related decisions and adjustments. Consequently, investigating students’ SWB is of substantial practical significance. SWB refers to an individual’s overall evaluation of their life, encompassing both emotional experiences and cognitive judgments of life satisfaction (Cui and Zhou, 2025). Regarding career development, evidence suggests that individuals with higher SWB are better equipped to handle academic and occupational stress and are at a lower risk of burnout (Liu et al., 2025; Safariningsih et al., 2024). Additionally, students with elevated SWB tend to display greater initiative in their work and maintain composure in social contexts (Lu et al., 2024).

Although the association between career exploration behavior (CEB) and SWB has been examined, research in this area remains limited, and the underlying mechanisms are not yet fully understood. According to career construction theory, individuals develop career adaptability (CA) through active career exploration, which enables them to adjust goals and strategies to navigate career-related uncertainties and is linked to greater overall SWB. Several studies have investigated the link between CEB and CA (Lu and Jia, 2022), however, few have examined whether CA functions as a mediating mechanism between CEB and SWB, particularly among university students. Meanwhile, perceived control, as a psychological resource, reflects individuals’ sense of agency in career decisions and future development. Career construction theory suggests that perceived control may strengthen the association between CEB and CA, yet the underlying mechanism has not been systematically explored (Zehui et al., 2025). Existing studies often focus on single dimensions or linear relationships and lack an integrated analysis of the interplay among CEB, CA, and perceived control. Guided by the framework of career construction theory (Savickas et al., 2009), the present study examines both the mediating role of CA and the moderating role of perceived control in the link between CEB and SWB, providing quantitative evidence from university students. This approach not only tests the core mechanisms proposed by career construction theory but also explores how psychological resources may moderate the link between career exploration and CA, thereby addressing gaps in prior research and offering useful advice for higher education career development and mental health interventions.

### 1.1 Theoretical framework

According to career construction theory, people mold their careers through personal narratives and self-reflection (Savickas et al., 2009). It conceptualizes career development as a continuous, evolving process, in which individuals construct a coherent career identity by meaningfully integrating past experiences with future aspirations. Importantly, this process of understanding and constructing one’s career occurs within specific social and cultural contexts. Through reflection and personal growth, individuals reinterpret their professional trajectories, enabling them to formulate career goals and cultivate the adaptive capacities necessary to navigate a dynamic work environment (Jiang et al., 2022; Savickas et al., 2009). Career construction theory further highlights how career goals and paths continuously evolve in response to changes in individual cognition and external circumstances, facilitating personal success and adaptation to the ever-changing workplace.

In this study’s theoretical model, CEB is regarded as a key antecedent that initiates the career construction process. According to career construction theory (Savickas et al., 2009), active engagement in CEB is linked to the accumulation of CA, which supports students in navigating external changes. This study further proposes that the CA accumulated through CEB is associated with higher SWB, forming a coherent theoretical chain of “exploration → adaptability → well-being” (Kara, 2024; Xiao et al., 2021). In addition, this study includes perceived control as a moderator to account for career construction theory’s emphasis on the interaction between individual agency and social context. Specifically, individuals with higher levels of perceived control are more likely to translate the information and experiences gained from CEB into CA, indicating that perceived control positively moderates the relationship between CEB and CA (Zehui et al., 2025). Through this moderated mediation model, the study not only examines the mediating role of CA but also identifies the individual psychological boundary conditions under which this pathway operates, thereby enhancing career construction theory’s explanatory power regarding the association between CA and SWB among university students.

### 1.2 Career exploration behavior and subjective well-being

CEB is a goal-directed, information-seeking activity in which individuals actively acquire information related to potential career paths. It represents a core component of career self-management (Yu et al., 2021). It is important to emphasize that CEB, as a behavioral construct, focuses on the actions individuals take to obtain career-related information. Empirical evidence indicates that CEB is positively associated with SWB (Ran et al., 2023). According to career construction theory, CEB is a deliberate and reflective process through which individuals examine past experiences, clarify personal goals, and progressively construct a coherent career identity (Savickas et al., 2009). Engagement in CEB can enhance self-confidence and reinforce the consistency of one’s career identity, which are associated with higher SWB (Chang et al., 2024; Gamboa et al., 2023). Ran et al. (2023) found that CEB can improve individuals’ SWB. Specifically, CEB reduces uncertainty regarding future career paths, alleviates career-related worries, and promotes goal clarity, thereby contributing to increased SWB (Zhang et al., 2022). Furthermore, CEB expands social networks and provides emotional support through interactions with peers or experts, and these social resources are also associated with higher SWB (Ouyang et al., 2025). Collectively, these findings highlight a positive association between CEB and SWB. This synthesis integrates prior research and offers a more systematic theoretical framework for understanding the link between CEB and well-being.

### 1.3 The mediating role of career adaptability

CA is a set of psychological tools that assist individuals in proactively managing career-related responsibilities, changes, and challenges in the workplace (Çarkıt, 2024). Unlike CEB, CA is a competency-level construct reflecting the capabilities individuals possess to adapt to changes in the career environment. Empirical evidence indicates that CA is positively associated with SWB (Tian et al., 2021). For instance, Kara (2024) found that higher CA are linked to enhanced SWB. People with elevated CA demonstrate greater capacity to cope with work-related changes and stress, exhibit increased emotional stability, and experience fewer negative impacts in the workplace, all of which contribute to higher SWB (Ouyang et al., 2025). Strong CA facilitates more effective adaptation to the work environment, increases coping efficiency, and promotes well-being (Ouyang et al., 2025; Takao and Ishiyama, 2021). Furthermore, CA encompasses the development of skills and the cultivation of self-confidence; successfully overcoming professional obstacles and achieving career goals generates a sense of accomplishment that directly enriches SWB (Montano, 2024). Collectively, these findings underscore the essential role of CA in improving SWB.

CEB is positively associated with CA (Ran et al., 2023). Lu and Jia (2022) found that engagement in CEB enhances an individual’s CA. Through CEB, university students gain a clearer understanding of their interests, skills, and career goals, which improves self-awareness and clarifies future career directions. This heightened self-awareness facilitates more effective adaptation to the work environment and job demands, thereby strengthening CA (Wang H. D. et al., 2024). CEB also fosters awareness of active learning and career development, motivating students to seek information, participate in internships, and enroll in training programs, all of which contribute to enhanced CA (Xiao et al., 2021; Zhou et al., 2024). Furthermore, CEB supports students in making career choices aligned with their interests and competencies and in setting clear career goals, providing direction for career development and further reinforcing CA (Zhao et al., 2021). Higher CA, in turn, has been shown to improve SWB (Ouyang et al., 2025). According to career construction theory, CEB provides experiences and reflective opportunities that help students cultivate CA. This adaptability enables them to adjust strategies, manage uncertainties, and construct a coherent career identity, which is linked to higher SWB. Collectively, these studies support a sequential pathway in which CEB is associated with CA, and increased CA is subsequently associated with higher SWB. Although previous research often examines the CEB–CA and CA–SWB relationships separately, an integrated analysis provides a clearer understanding of the process and a more coherent theoretical foundation for positioning CA as a mediator between CEB and SWB.

### 1.4 The moderating role of perceived control

Perceived control indicates a person’s confidence in their capacity to control career-related outcomes and effectively cope with challenges during career exploration (Batucan et al., 2025). Unlike career CEB at the behavioral level and CA at the competency level, perceived control represents a cognitive-level psychological resource, reflecting individuals’ subjective beliefs about their ability to manage their career development. According to career construction theory, higher perceived control makes it easier for people to integrate exploration experiences, translate them into CA, and develop a coherent career identity (He et al., 2026). Individuals with elevated perceived control typically perceive greater agency over their career choices and future paths. During the career exploration process, they demonstrate stronger initiative and motivation, coupled with greater confidence in addressing career challenges, which in turn enhances CA (Zehui et al., 2025). Perceived control also facilitates emotional regulation and promotes a positive mindset during exploration, contributing to the development of CA (Deniz, 2026). This study positions perceived control as a moderator rather than a mediator, and this distinction has a clear theoretical basis. While CEB provides experiences and opportunities for reflection that directly contribute to CA, perceived control plays a key moderating role by shaping the depth and strategic orientation of exploration, as well as the extent to which exploration experiences are translated into CA (Wang L. et al., 2024). In other words, perceived control alters the strength or direction of the relationship between CEB and CA, rather than serving as a mechanism transmitting this relationship. Individuals with higher levels of perceived control can derive greater benefits from the same CEB, such that the positive association between CEB and CA is stronger for those with elevated perceived control. By defining this moderating role, the study clearly separates the main-effect pathway of “behavior → ability” from the moderating pathway of cognitive beliefs as a boundary condition, thereby eliminating potential conceptual ambiguity between variables. Collectively, CEB, CA, and perceived control constitute a dynamic and interactive developmental system, clearly illustrating the moderating role of perceived control in CA development and highlighting the importance of psychological resources in linking career exploration to adaptability.

### 1.5 This study

**Figure 1:**
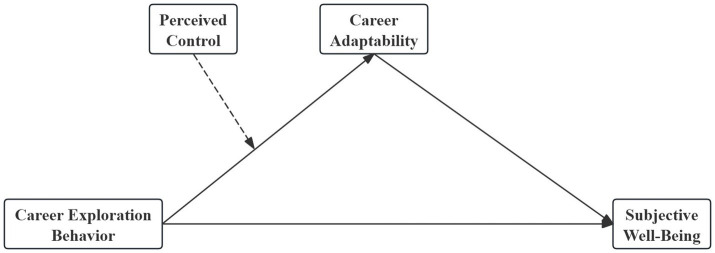
Study model.

University students face multiple challenges, including academic pressure and career planning. However, existing research has not thoroughly examined how CEB is linked to CA and SWB. Moreover, perceived control, which may shape the relationship between CEB and CA, remains insufficiently examined. Drawing on career construction theory, the present study proposes a moderated mediation model to analyze whether CEB is associated with students’ SWB through CA and to examine the moderating role of perceived control. This model serves two purposes: to clarify the relationship between CEB and SWB; and to explore how perceived control moderates the association between CEB and CA. This theoretical framework offers a basis for comprehending mechanisms that can support improvements in students’ SWB. Figure 1 depicts the overall research model. Based on this model, the following hypotheses are proposed:

*H1*: CEB is significantly positively correlated with SWB.

*H2*: CEB is significantly positively correlated with CA.

*H3*: CA is significantly positively correlated with SWB.

*H4*: CA mediates the link between CEB and SWB.

*H5*: Perceived control moderates the link between CEB and CA.

## 2 Methods

### 2.1 Participants and procedure

This study was conducted from October to November 2025 using a convenience sampling method to recruit participants from three universities in China. The research team first contacted the relevant departments at each university to explain the study purpose and participation requirements. The departments then assisted in distributing invitations to eligible students via email or class group messages, and these invitations included the questionnaire link and participation instructions. All students who received the invitation were free to decide whether to participate, and those who agreed completed the online questionnaire through the Wenjuanxing platform. Prior to participation, students read and signed an electronic informed consent form to ensure voluntary and informed participation. The study strictly adhered to confidentiality principles, and all data were collected solely for academic research purposes.

The survey consisted of 63 items. Following Kline (2018) sample size guidelines, the required baseline sample was 630 participants. Considering a potential 20% rate of invalid or incomplete responses, the target sample size was set at 756 participants (63 × 10 + 20% × 63 × 10). A total of 882 questionnaires were collected, of which 65 were excluded due to invalid responses, including questionnaires with more than 20% missing answers or over 80% of items marked as either “very consistent” or “very inconsistent.” Consequently, 817 valid surveys were kept, resulting in a 92.63% effective response rate. The largest subgroup comprised senior students, who accounted for 48.1% of the sample (Table 1).

**Table 1:** Demographic information.

Demographic characteristics	Category	Number	Percentage (%)
Gender	Male	345	42.2
	Female	472	57.8
Age	18–20 Years	245	30.0
	21–22 Years	447	54.7
	Over 22 Years	125	15.3
Grade	Freshman	128	15.7
	Sophomore	142	17.4
	Junior	154	18.8
	Senior	393	48.1

### 2.2 Measurement tools

#### 2.2.1 Career exploration behavior

The CEB Scale was used in this investigation, originally created by Stumpf et al. (1983) and confirmed by Xu (2008). The scale is designed to assess university students’ engagement in CEB and consists of 18 items covering four dimensions: environmental exploration, self-exploration, goal-system exploration, and information-seeking. An example item is, “I understand various job paths connected to my field of study.” A 5-point Likert scale is used to grade responses; higher overall scores indicate more engagement in career exploration. Strong validity and reliability have been shown by the CEB Scale in Chinese samples (Ruan et al., 2025). Its Cronbach’s *α* was 0.946.

#### 2.2.2 Career adaptability

The CA Scale, which was originally developed by Savickas (1997) and confirmed by Hou et al. (2012), was used in this investigation. The scale is designed to assess university students’ CA and comprises 24 items across four dimensions: career confidence, career curiosity, career concern, and career control. “I make plans to reach my career aims.” is one example. A 5-point Likert scale is used to grade responses; greater overall scores indicate stronger CA. Within Chinese samples, the CA Scale has shown excellent validity and reliability (Jilong et al., 2024). Its Cronbach’s *α* was 0.957.

#### 2.2.3 Perceived control

This study employed the Sense of Control Scale to assess university students’ perceptions of personal control. The scale comprises 12 items covering two dimensions: personal control and perceived constraints. An example item is, “I can almost always finish anything I decide to do.” Responses are rated on a 7-point Likert scale, with higher total scores indicating a stronger sense of perceived control. The scale has demonstrated good reliability and validity among Chinese samples (Wang Z. et al., 2024). Its Cronbach’s α was 0.936.

#### 2.2.4 Subjective well-being

This study employed the Happiness Index Scale, developed by Campbell (1976), to assess university students’ SWB. The scale comprises 9 items, including an overall emotional index with 8 items and a single item measuring life satisfaction. An example item is, “I think life is enjoyable.” A 7-point Likert scale is used to rate responses. The average score of the overall emotional index (weighted 1.0) and the life satisfaction score (weighted 1.1) are combined to create the total happiness index, which can range from 2.1 (least happy) to 14.7 (most happy), with scores higher denoting greater SWB. In Chinese samples, the scale has shown strong validity and reliability (Su and He, 2023). Its Cronbach’s α was 0.894.

### 2.3 Data analysis

Data analysis was conducted using SmartPLS 4.0. Partial least squares structural equation modeling (PLS-SEM) was employed to examine the relationships among CEB, CA, perceived control, and SWB. The study model comprised four latent variables and 63 measurement questions, based on 817 verified surveys. Given the complexity of the structural model, the use of SmartPLS 4.0 was appropriate and provided a robust analytical performance.

## 3 Results

### 3.1 Descriptive statistics and correlation analysis

Correlation analysis indicated that CEB was strongly and positively linked to CA (*r* = 0.501, *p* < 0.001) and SWB (*r* = 0.557, *p* < 0.001). CA was strongly and positively linked to SWB (*r* = 0.498, *p* < 0.001) (Table 2), this indicates that students with higher CEB generally show greater CA and higher SWB.

**Table 2:** Descriptive statistics and correlation analysis.

Construct	M ± SD	CEB	CA	PC	SWB
CEB	3.072 ± 1.009	1			
CA	3.139 ± 0.979	0.501***	1		
PC	4.183 ± 1.622	0.382***	0.409***	1	
SWB	9.231 ± 3.038	0.557***	0.498***	0.574***	1

CEB: career exploration behavior, CA: career adaptability, PC: perceived control, SWB: subjective well-being. *** denotes a significance level of *p* < 0.001.

### 3.2 Common method bias (CMB)

Harman’s single-factor test revealed five factors with eigenvalues larger than 1. The first factor explained 32.726% of the variance overall, which is less than the 40% threshold commonly considered indicative of potential CMB (Podsakoff and Organ, 1986).

In addition, the common latent factor approach was employed to further evaluate CMB. A latent factor was introduced to the measurement model and linked to every observed indicator. The original model, which did not include this latent factor, was then compared with the modified model (Podsakoff et al., 2003). Results indicated no statistically meaningful difference between the original and modified models (*p* > 0.05). Taken together, the findings from both approaches suggest that the risk of common method bias in this study was minimal.

### 3.3 Measurement model

The measurement model’s validity and reliability were thoroughly evaluated in accordance with Hair Jr et al. (2021). Reliability was evaluated using item factor loadings and composite reliability (CR). Generally, item loadings should exceed 0.70. Although a few items had loadings below this threshold, they were retained because the CR and average variance extracted (AVE) met acceptable standards (Table 3). CR was employed to assess internal consistency, with a recommended cutoff of 0.70. The scales demonstrated strong item loadings and high CR (Table 3), indicating good reliability. Discriminant validity was assessed using both the Heterotrait-Monotrait Ratio (HTMT) and the Fornell-Larcker criterion. All HTMT values were below the recommended cutoff of 0.90 (Table 4), and the correlations between constructs were less than the square root of their respective AVE (Table 5), satisfying the Fornell-Larcker criterion. Collectively, these results show that the study’s latent constructs have strong reliability and validity (Badwy et al., 2025).

**Table 3:** Reliability and validity of the model.

Constructs	Items	Loadings	Cronbach’s alpha	CR	AVE
CEB	CEB1	0.672	0.946	0.951	0.521
	CEB2	0.702			
	CEB3	0.734			
	CEB4	0.728			
	CEB5	0.734			
	CEB6	0.731			
	CEB7	0.741			
	CEB8	0.720			
	CEB9	0.730			
	CEB10	0.732			
	CEB11	0.728			
	CEB12	0.713			
	CEB13	0.720			
	CEB14	0.716			
	CEB15	0.738			
	CEB16	0.701			
	CEB17	0.733			
	CEB18	0.718			
CA	CA1	0.712	0.957	0.960	0.502
	CA2	0.715			
	CA3	0.711			
	CA4	0.717			
	CA5	0.696			
	CA6	0.711			
	CA7	0.708			
	CA8	0.704			
	CA9	0.712			
	CA10	0.721			
	CA11	0.702			
	CA12	0.710			
	CA13	0.716			
	CA14	0.719			
	CA15	0.712			
	CA16	0.718			
	CA17	0.711			
	CA18	0.700			
	CA19	0.699			
	CA20	0.712			
	CA21	0.702			
	CA22	0.701			
	CA23	0.704			
	CA24	0.695			
SWB	SWB1	0.748	0.894	0.913	0.540
	SWB2	0.747			
	SWB3	0.749			
	SWB4	0.717			
	SWB5	0.742			
	SWB6	0.758			
	SWB7	0.717			
	SWB8	0.710			
	SWB9	0.723			

**Table 4:** HTMT criteria.

Construct	CA	CEB	SWB
CA			
CEB	0.527		
SWB	0.507	0.553	

**Table 5:** Fornell-Larcker criteria.

Construct	CA	CEB	SWB
CA	***0.709***		
CEB	0.504	***0.722***	
SWB	0.478	0.519	***0.735***

On the table’s diagonal, the square root of AVE is shown in bold, italicized style.

### 3.4 Structural model

#### 3.4.1 Collinearity test

According to empirical guidelines, variance inflation factor (VIF) values should be below 3.3. As shown in Table 6, VIF values in this study ranged from 1.000 to 1.341, indicating that there was no significant multicollinearity in the model. This suggests that the model coefficients were stable and that the relationships among variables could be interpreted reliably.

**Table 6:** VIF.

Construct	CA	CEB	SWB
CA			1.341
CEB	1.000		1.341
SWB			

#### 3.4.2 Path hypotheses

**Figure 2:**
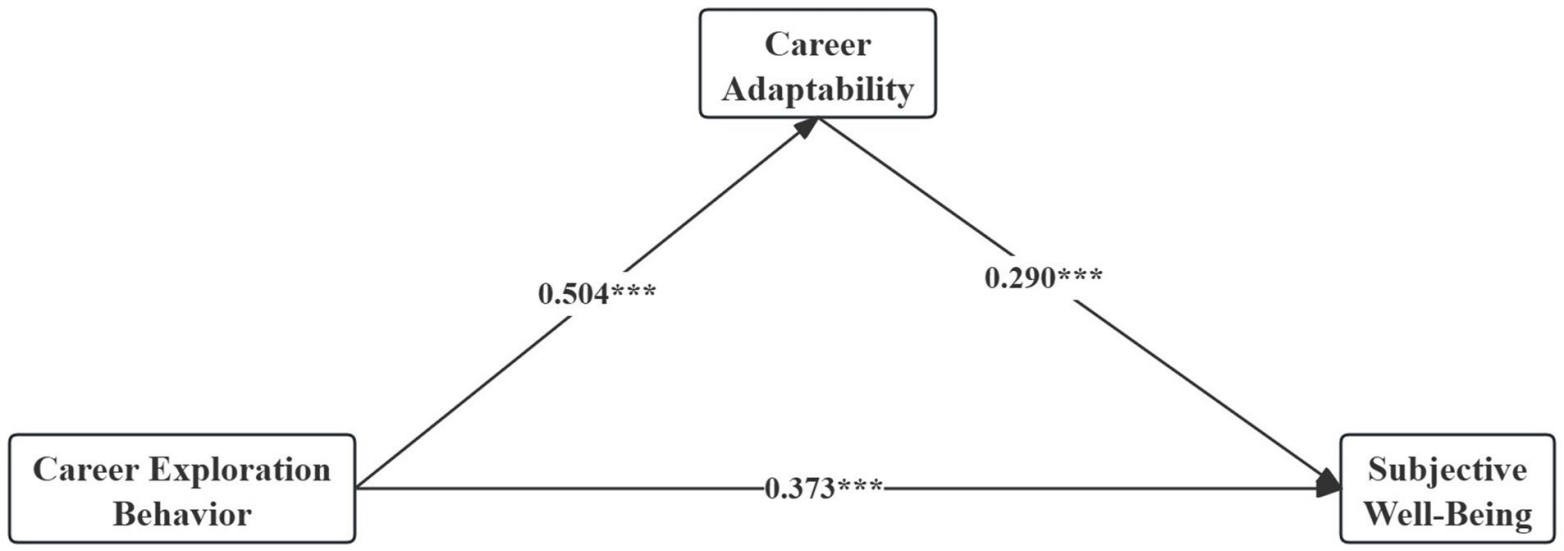
Path diagram.

In the structural model analysis, PLS-SEM was used, and path coefficients and their significance were estimated using 5,000 bootstrap samples (Qalati et al., 2025; Qalati et al., 2026). The findings showed a strong positive association between CEB and SWB (*β* = 0.373, *t* = 10.887, *p* < 0.001) (Table 7; Figure 2). These findings suggest that students who actively engage in CEB may develop a better understanding of their interests and CA, enhance self-confidence, and experience higher SWB.

**Table 7:** The mediation models of CA in the relationship between CEB and SWB (*n* = 817).

Path	*β*	*t*	2.5%	97.5%	*p*	Result
CEB → SWB	0.373	10.887	0.308	0.441	< 0.001	Supported
CEB → CA	0.504	16.983	0.447	0.565	< 0.001	Supported
CA → SWB	0.290	9.439	0.228	0.351	< 0.001	Supported

#### 3.4.3 Coefficient of determination

The structural model accounted for a moderate proportion of variance in SWB, with R^2^ = 0.332 (Table 8), indicating that CEB and CA effectively capture variations in SWB. In addition, every Q^2^ value was higher than zero (Table 8), demonstrating that the model had substantial predictive relevance.

**Table 8:** Explanatory power and predictive relevance.

Construct	R^2^	Q^2^	Model fit
CA	0.254	0.126	SRMR:0.043
SWB	0.332	0.172	NFI:0.882

### 3.5 Mediation analysis

CEB exhibited a significant direct association with SWB (*β* = 0.373, *t* = 10.887, *p* < 0.001). In addition, the mediating effect of CA was also significant (*β* = 0.146, *t* = 8.781, *p* < 0.001) (Table 9), indicating that CA partially mediated the relationship between CEB and SWB.

**Table 9:** Mediation effects of CA.

Path	Indirect effect	*t*	*p*	Direct effect	*t*	*p*	Mediation type
CEB → CA → SWB	0.146	8.781	< 0.001	0.373	10.887	< 0.001	CPM

CPM, complementary partial mediation.

### 3.6 Moderation analysis

The two-stage method was employed to investigate the moderating impact of perceived control on the link between CEB and CA. The two-stage method is frequently employed in moderation analyses and is thought to be more accurate than both the product indicator and orthogonal methods (Becker et al., 2018; Henseler and Chin, 2010). R^2^ for CA was 0.254 after the interaction term was removed from the model, meaning that 25.4% of the variance in CA was explained by the model. After including the interaction term between CEB and perceived control, R^2^ rose to 0.324, indicating a 7.0% increase in explanatory power. This demonstrates that the inclusion of perceived control as a moderator enhances the model’s explanatory capability. Perceived control significantly moderated the link between CEB and CA, as evidenced by the strong positive association between CEB and CA (*β* = 0.386, *t* = 10.461, *p* < 0.001) and the significant interaction term (*β* = 0.112, *t* = 4.070, *p* < 0.001) (Table 10).

**Table 10:** Moderation analysis.

Path	*β*	*SE*	*t*	Bootstrap 95% CI		*p*
				LLCI	ULCI	
CEB → CA	0.386	0.037	10.461	0.313	0.458	< 0.001
PC × CEB → CA	0.112	0.028	4.070	0.059	0.165	< 0.001

**Figure 3:**
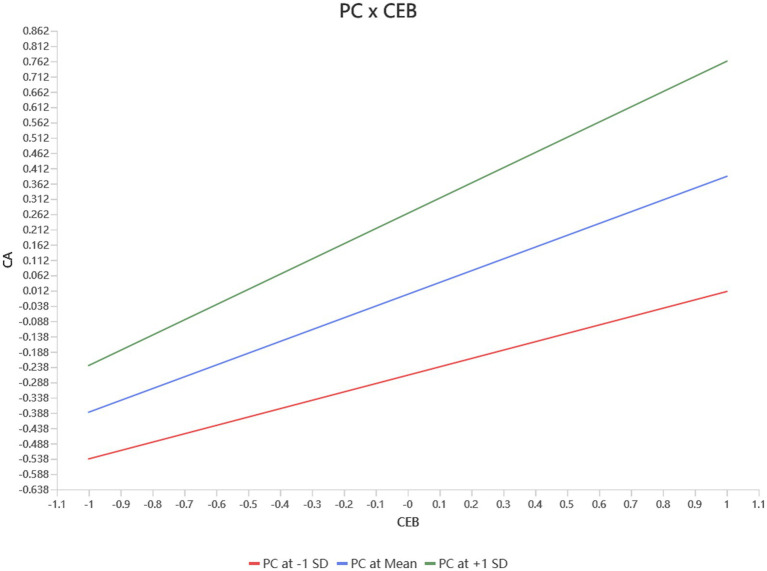
Simple slope analysis.

To further examine the nature of the moderating effect, a simple slope analysis was conducted (Figure 3). The findings showed that perceived control significantly moderates the link between CEB and CA. In particular, the positive association between CEB and CA was strongest at high levels of perceived control (+1 SD) and weakest at low levels (−1 SD). These findings suggest that higher perceived control amplifies the benefits of CEB on CA, particularly when CEB is at a high level. Overall, the findings confirm the positive moderating effect of perceived control and underscore the potential value of enhancing individuals’ perceived control to maximize the effectiveness of CEB in career development practice.

## 4 Discussion

CEB and SWB exhibited a significant positive correlation, thereby supporting H1. Consistent with Ran et al. (2023), higher levels of CEB enhance individuals’ SWB and assist university students plan their career paths. According to career construction theory (Savickas et al., 2009), individuals continuously adjust the fit between their abilities and external opportunities through ongoing career exploration. This process not only helps students plan their career paths more effectively but also strengthens their confidence and motivation in coping with career uncertainty, which is associated with higher SWB (W. Ouyang et al., 2025; Zhang et al., 2022). In China’s highly competitive and uncertain employment environment, active career exploration is particularly important, as it helps students maintain perceived control and psychological resources, supporting their SWB (Chang et al., 2024). These findings indicate that CEB is not only an important correlate of career planning and SWB but also theoretically supports career construction theory’s emphasis on the central role of proactive exploration in career development. At the practical level, universities can support students’ career exploration and the accumulation of psychological resources through structured activities such as internships, career planning courses, and mentorship programs, thereby fostering their SWB and career development capabilities.

CA functioned as a mediator in the link between CEB and SWB, thereby supporting H2, H3 and H4. According to career construction theory, individuals match their personal traits with the career environment through exploring career options, gradually forming a coherent career self-narrative, which provides a psychological foundation for the development of CA (Savickas et al., 2009). Students with higher CA typically show greater confidence and a more optimistic outlook, allowing them to adjust goals and cope with stress more effectively, which is associated with higher SWB (Montano, 2024). In contrast, students with lower CA tend to experience greater uncertainty and anxiety in career decision-making, which is associated with lower SWB (Takao and Ishiyama, 2021). The quantitative analysis in this study further indicates that CA mediates the relationship between CEB and SWB through psychological regulation and adaptive mechanisms. In China’s highly competitive and uncertain employment context, enhancing CA is particularly helpful for students in managing internship choices, career decisions, and future job-related stress. This also highlights the practical value of universities supporting students’ psychological resources through activities such as career simulations. Overall, the mediating role of CA aligns with the core assumptions of career construction theory, providing both theoretical and empirical support for understanding the relationship between CEB and SWB, while also highlighting its practical relevance in China’s specific career context.

This research revealed that perceived control moderates the link between CEB and CA, thereby supporting H5. This result aligns with He et al. (2026), which indicates that self-control reduces emotional conflict during career exploration, thereby facilitating the development of CA. According to career construction theory (Savickas et al., 2009), perceived control is a psychological resource that can enhance students’ ability to effectively match their personal traits with the career environment during career exploration. During career exploration, students with higher perceived control show greater confidence in handling uncertainty, actively engage in career exploration, and exhibit higher CA (Zehui et al., 2025). In contrast, students with lower perceived control are more likely to feel helpless and anxious, participate less in career exploration, and demonstrate lower CA (Deniz, 2026). From a psychological mechanism perspective, perceived control strengthens the relationship between CEB and CA by enhancing self-efficacy, resource perception, and the sense of controllability over environmental challenges. These findings not only provide empirical support but also align with the core assumptions of career construction theory, which posit that university students develop CA through proactive exploration, and that perceived control, as a psychological resource, can strengthen this process, enhancing their sense of control over career paths and psychological fulfillment. In China’s highly competitive and uncertain employment context, enhancing perceived control is particularly helpful in supporting students’ career exploration and CA, offering practical guidance for university career counseling and educational interventions.

## 5 Significance and limitations of the study

### 5.1 Theoretical significance

Based on career construction theory, this study examined the relationship between university students’ CEB and SWB, analyzed the mediating role of CA, and tested the moderating effect of perceived control. The study offers three primary theoretical insights. First, it empirically validates the mediating role of CA between CEB and SWB, providing quantitative evidence for the core theoretical premise that students enhance well-being through active career construction. This offers a more precise assessment of the psychological mechanism linking career exploration to well-being. Second, the study incorporates perceived control into the link between CEB and CA and confirms its moderating role. Students with higher perceived control exhibit a stronger positive association between CEB and CA, highlighting the significance of individual psychological resources in career development and addressing a gap in prior research that overlooked individual differences. Third, the study integrates CEB, CA, and perceived control within a single framework, illustrating how active career behaviors, internal adaptability, and control beliefs jointly shape psychological outcomes. This provides a theoretically coherent interaction model and offers guidance for future study and intervention design. Overall, the study not only validates key aspects of career construction theory but also extends its empirical and practical significance by clarifying the underlying mechanisms and boundary conditions of career exploration and psychological well-being.

### 5.2 Practical significance

From the perspective of higher education institutions, this study demonstrates that CEB is positively correlated with SWB through CA, and that perceived control moderates the relationship between CEB and CA. Based on these findings, universities can implement targeted strategies to enhance students’ CA and perceived control. Such strategies may include structured career exploration activities (e.g., internships, simulated workplace scenarios, and goal-setting workshops), as well as training in decision-making and coping with uncertainty and reflective career counseling. These interventions can increase students’ engagement in career planning and strengthen the positive effects of CEB on both CA and SWB.

From the perspective of individual students, perceived control constitutes a key psychological resource in career exploration. Students can enhance their perceived control by clarifying career goals, engaging in diverse career experiences, and applying self-regulation strategies, such as planning, reflection, and stress management. Participation in structured career exploration activities can further develop CA, which is linked to increased SWB. Based on these findings, students are encouraged to plan their career paths in detail, adjust their expectations according to their progress, and focus on opportunities that align with their interests and abilities, thereby maximizing the psychological benefits of career exploration.

### 5.3 Limitations and future research directions

There are various restrictions. First, a cross-sectional design was utilized, with data collected at a single time point, which limits the ability to analyze changes in variables over time and precludes causal inferences. Future research could adopt longitudinal or multi-wave designs to examine the dynamic relationships and potential causal pathways among CEB, CA, perceived control, and SWB. Second, this study relied on self-report questionnaires, which may introduce social desirability or response biases, potentially affecting the robustness of the results. To address CMB, both Harman’s single-factor test and a latent variable approach were used to ensure methodological rigor. Future studies could incorporate multi-source measurements, behavioral observations, or interviews to further enhance reliability and generalizability. Finally, the results may have limited generalizability to other cultural situations, as the sample was mainly composed of Chinese university students. Future research might increase the sample size to include students from various regions and universities, thereby providing a broader perspective. Comparing CEB and its relationship with SWB across cultural backgrounds would facilitate cross-cultural research on career development and increase the applicability of findings to diverse contexts.

## 6 Conclusion

Based on career construction theory, this study assessed the link between CEB and SWB, analyzed the mediating role of CA, and tested the moderating effect of perceived control. The results showed a significant positive correlation between CEB and SWB, with CA partially mediating this relationship. Additionally, perceived control significantly moderated the link between CEB and CA, with a greater positive association when perceived control is higher. From a theoretical standpoint, this study expands the application of career construction theory by introducing CA and perceived control as a research variable and clarifying the psychological mechanisms linking career exploration to well-being. Practically, the findings offer guidance for career development initiatives in higher education, suggesting that universities should encourage active career exploration and prioritize interventions that enhance students’ CA and perceived control to improve their SWB. Nevertheless, the study’s cross-sectional design and reliance on self-report measures, which could lead to response biases, are among its limitations. Future studies may use longitudinal designs and multiple data collection methods to further validate these findings and ensure the stability and reliability of the results.

## Data Availability

The raw data supporting the conclusions of this article will be made available by the authors, without undue reservation.
